# Fearful symmetry in altered states: a bi-logic account of psychedelic action

**DOI:** 10.3389/fpsyg.2025.1632923

**Published:** 2025-08-19

**Authors:** Matthew Goldreich

**Affiliations:** Department of Psychology, University of Exeter, Exeter, United Kingdom

**Keywords:** psychedelics, altered states, logic, symmetry, psychoanalysis, philosophy of mind, Ignacio Matte Blanco, Sigmund Freud

## Abstract

This conceptual study examines Matte Blanco’s system of bi-logic as a novel framework for understanding psychedelic altered states of consciousness. The initial point of departure is a consideration of the complex historical relationship between psychoanalysis and psychedelics, prompting a review of contemporary psychoanalytic and neuropsychoanalytic perspectives on psychedelic action. This leads into an exposition of bi-logic, which reformulates Freud’s conception of conscious and unconscious processes in terms of logico-mathematical principles, postulating binary modes of mental functioning: the *asymmetrical mode of being*, characterized by logic, differentiation, ordered relations in space and time, and cognition; and the *symmetrical mode of being*, characterized by symmetry, generalization, unity, spacelessness, timelessness, paradox, and boundless affect. The ‘bi-logic account’ elaborated here posits that psychedelic altered states tend towards the symmetrical mode of being: psychedelics shift the balance between these modes such that the symmetrical mode is increasingly prevalent in subjective experience and influential in mental functioning compared with ordinary states of consciousness. Having delineated this framing, its heuristic value in relation to the therapeutic use of psychedelics is discussed, particularly in the context of psychoanalytically-informed psychedelic therapy. Bi-logic is anticipated to contribute to a conceptual container which facilitates greater receptivity to, and integration of, experiences marked by intense affect, paradox, and the dissolution of self-other boundaries.

## 1 Introduction

Tyger Tyger, burning bright,

In the forests of the night;

What immortal hand or eye,

Could frame thy fearful symmetry?

–William Blake, *The Tyger*

Drawing upon his clinical work with psychotic patients, Matte Blanco (1975) made an important contribution to psychoanalytic thought by reformulating Freud’s conception of conscious and unconscious processes in terms of logico-mathematical principles. In particular, Matte Blanco builds upon Freud’s intuition that each mode of thought is governed by a distinct form of logic: “the logical laws of thought do not apply in the id” (Freud, 1933, p. 73, 1900, 1911, 1915). Matte Blanco elucidates a distinct set of logical principles governing each mode: a system of *bi-logic.* In brief, the *asymmetrical mode of being* is characterized by logic, differentiation, ordered relations in space and time, and cognition; whilst the *symmetrical mode of being* is characterized by symmetry, generalization, unity, spacelessness, timelessness, paradox, and boundless affect. An important aspect of bi-logic is that although these modes are conceived of as binary, their underlying processes are fluid and continuous - any given psychical state exhibits a varying degree of both modes of mental functioning. This framework is described extensively below in the section “*A bi-logic account of psychedelic action*.”

This conceptual study examines Matte Blanco’s metapsychology as a novel framework for understanding psychedelic altered states of consciousness. The initial point of departure is a consideration of the complex historical relationship between psychoanalysis and psychedelics, prompting a review of contemporary psychoanalytic and neuropsychoanalytic perspectives on psychedelic action. Although we will find a significant emphasis in this literature on the amplification of psychodynamic processes under psychedelics, the logical characteristics of these processes have not yet been unpacked in relation to psychedelic action. Rundel (2022) opens the way for this inquiry when she states that Matte Blanco’s bi-logic

is an important link between psychoanalysis and psychedelics in that it offers a theoretical understanding of united states beyond the personal unconscious, toward a felt sense of connection with all beings and all of reality, a transpersonal layer of consciousness that is often a feature of psychedelic understanding. (Rundel, 2022, p. 473)

The aim of the present study is to offer a conceptual framing of this ‘important link’ and to discuss the clinical implications of the theoretical understanding which emerges. In brief, the ‘bi-logic account’ elaborated here posits that psychedelic altered states increasingly tend towards the symmetrical mode of being compared with ordinary states of consciousness. By this account, psychedelic action can be conceived of in terms of a shift in the balance between these modes such that the symmetrical mode is increasingly prevalent in subjective experience and influential in mental functioning. This novel framing is anticipated to contribute heuristic value to the therapeutic use of psychedelics, particularly in the context of psychoanalytically-informed psychedelic therapy.

## 2 A brief history of psychedelic psychoanalysis

The initial encounter between psychoanalysis and psychedelics began in the 1950s, an era in which psychoanalysis had reached the peak of its influence in psychiatry and culture. At this time, clinical research on a set of newly synthesized compounds - then grouped together by the name *psychotomimetics* (meaning ‘psychosis mimicking’) - was entering into its golden age (Osmond, 1957; Guss, 2022). Medical psychotherapists, generally from a psychoanalytic orientation, began clinical trials of *psycholytic* (low dose) therapy using LSD and psilocybin, initially with psychotic patients and later with neurotic patients also (e.g., Busch and Johnson, 1950; Eisner and Cohen, 1958; Sandison et al., 1954; Chandler and Hartman, 1960; Passie et al., 2022).

Given the pre-eminence of psychoanalysis during this epoch, early formulations of psychedelic altered states in the psychiatric literature inevitably conform with a psychoanalytic metapsychology. Construing their findings through a psychoanalytic lens, these clinicians observe the modulation of a range of interpersonal and intrapsychic mechanisms: the reduction of ego defenses, increased association and symbolic function, an intensification of transference relations, decreased resistance to interpretations, and *abreaction*, i.e., the recall and relieving of repressed memories through regression to early relational states (e.g., Busch and Johnson, 1950; Eisner and Cohen, 1958; Sandison et al., 1954; Chandler and Hartman, 1960; Abramson, 1967; Leuner, 1967; Buckman, 1967).

Altered states of consciousness have played an integral role in the historic development of psychoanalysis, which was at its outset borne out of Breuer and Freud’s (1893) observations in their work with hypnosis and Freud’s (1900) interpretation of the altered states of dreaming and psychosis (Rundel, 2022; Carhart-Harris and Friston, 2010). This is to say that psychoanalysis is and always has been informed by revelation in the form of altered states to conceptualize unconscious processes (Aron and Bushra, 1998). Thus, if psychedelics are *mind revealing* [this is the etymology of the term ‘psychedelic’ (Osmond, 1957)], and psychoanalytic inquiry relates to these same depths, common ground is expected here. These early psycholytic studies are characterized by the amplification of interpersonal and intrapsychic processes, conforming with and providing support for existing psychoanalytic theory.

Following the initial period of psycholytic trials in Europe, clinicians in North America began experimenting with high dose sessions, which they termed ‘psychedelic therapy’ (Osmond, 1957; Pahnke, 1963; Grof, 1973; Walsh and Grob, 2006). As described above, it had been thought that psycholytic therapy sessions “fostered the amplification and exploration of psychodynamic issues.” Conversely, high-dose sessions “tended to quickly catapult subjects through the psychodynamic levels and on to transpersonal and even mystical experiences” (Walsh and Grob, 2006, p. 435). Researchers began to view ‘psychodynamic issues’–the interpersonal and intrapsychic–as transcended at higher doses, the subject’s experience intensifying (or regressing) from developmental to perinatal^1^ to transpersonal, from personal to collective unconscious (Grof, 1973, 2009; Walsh and Grob, 2006). Such altered states soon became the intended direction of these sessions (Leuner, 1967; Passie et al., 2022). Accordingly, as the clinical interest in high dose sessions began to increase, the influence of psychoanalysis in psychedelic clinical trials began to diminish.

When psychedelics began to escape the lab in the 1960s, psychedelic research became increasingly regulated, and these substances began to lose their identity as novel medicines with promise for treating a range of psychiatric disorders (Guss, 2022). Whilst the influence of psychedelics proliferated in countercultural spiritual, artistic, and intellectual movements, they came to be viewed in the wider culture as recreational drugs liable to abuse, becoming associated with damaging countercultural slogans such as “turn on, tune in, drop out” (Leary, 1965). A stream of misinformation about the dangers of psychedelics broke through into public consciousness, including images of brains being fried like eggs and myths of chromosome damage, which were later falsified (e.g., Dishotsky et al., 1971). This culminated when the United States enacted The Controlled Substances Act of 1970, the United Kingdom following suit with the Misuse of Drugs Act 1971, enshrining the war on drugs into law (Guss, 2022; Sessa, 2018). This effected a global ban not only on recreational use but also on clinical studies of these substances. As psychedelic research fell dormant, the cultural and societal impact of psychedelics went underground (Carhart-Harris and Goodwin, 2017).

Psychoanalysis, with its enmeshment in institutional psychiatry in North America, followed the law, not the counterculture, disavowing the role of psychoanalysis in psychedelic history and the role of psychedelics in psychoanalytic history, treating psychedelic substances with the suspicion and paranoia which had entered the mainstream discourse (Guss, 2022). In the context of contemporary psychoanalysis, Guss (personal communication) observes a growing openness to and curiosity about psychedelic therapy amongst many psychoanalytic clinicians, alongside a more resistant or guarded attitude in others, who view psychedelic use as dangerous, excessive, inauthentic, and childish.

The 21st Century has borne witness to a rapid expansion of scientific and cultural interest in psychedelics, eclipsing the classical era of psychedelic studies in the mid-20th Century: a so-called ‘psychedelic renaissance’^2^ (Sessa, 2018). In a clinical context, this has taken the form of a plethora of new studies investigating the safety and efficacy of psychedelics in treating issues ranging from treatment-resistant depression (e.g., Carhart-Harris et al., 2016; 2021); depression and anxiety associated with late-stage cancer diagnoses (e.g., Grob et al., 2011; Ross et al., 2016; Griffiths et al., 2016); addiction (e.g., Morgan et al., 2017; Johnson et al., 2014; Zafar et al., 2023); PTSD (e.g., Krediet et al., 2020; Henner et al., 2022); palliative care (Yaden et al., 2021); and improving well-being (Gandy, 2019). The recognition of these studies has resulted in a move towards decriminalization–in certain jurisdictions of the United States and Australia–with the process already underway for other states and countries to follow suit.

More than half a century after the schism between psychoanalysis and psychedelics, psychoanalytically oriented researchers have begun to re-engage with these substances, conceptualizing their mechanism of action through the lens of psychoanalytic and neuropsychoanalytic theory, and considering their integration into psychoanalytic clinical practice (e.g., Guss, 2022; Rundel, 2022; Fischman, 2019, 2022; Buchborn et al., 2023; Lichtenstein and Hoeh, 2024; Barrett, 2022; Passie et al., 2022). This contemporary literature advances psychoanalytic understanding of psychedelic altered states, incorporating an array of novel clinical and neuropsychoanalytic studies which are detailed in the following sections.

## 3 Psychedelics, primary process mentation, and symmetrical logic

Early observations of the intensification of psychodynamic processes under psychedelics can be understood to demonstrate the general manifestation of primary process mentation. In elaborating his drive theory, Freud observed that unconscious and conscious processes are governed by distinct forms of psychical functioning. He denoted these as the *primary* and *secondary process*, respectively (Freud, 1900, 1911, 1915).^3^ The primary process follows the *pleasure principle*, which seeks satisfaction of affective and somatic instinctual drives through the discharge of unbound (free) energy, even by means of hallucinatory wish-fulfilment (Bazan, 2023; Carhart-Harris and Friston, 2010). Conversely, the secondary process is governed by the *reality principle*, which demands adaption to the external world based on planning and conscious reflection. Its function is a regulation of the primary process through the conversion of free energy into bound energy through *cathexis*, i.e., investment into objects. At the affective level of primary process mentation, competing impulses, which would conflict in conscious thought, are able to co-exist. There is “no negation, no dubiety, no varying degree of certainty” (Freud, 1915, p. 134). Primary process mentation does not abide by relations in space or time, and psychic reality holds primacy over external reality. In contrast, the secondary process is the cognitive mode, entailing differentiation and ordered relations in time and space.

Perhaps the most compelling evidence for these dual modes of psychical functioning is altered states of consciousness which include, in addition to psychedelic states, dreaming, temporal lobe epilepsy, and psychosis (Carhart-Harris and Friston, 2010; Kräehenmann et al., 2017). These states are correlated with unconstrained primary process mentation (and with each other) at both a phenomenological and neuroscientific level of analysis (Carhart-Harris and Friston, 2010). Several studies using psychometric tests have demonstrated that primary process mentation is intensified under psychedelic use (Landon and Fischer, 1970; Martindale and Fischer, 1977; Natale et al., 1978; Kräehenmann et al., 2017). Kräehenmann et al. (2017) found that LSD increased levels of ‘cognitive bizarreness’, depth of affect, free association, and dreamlike and symbolic imagery, all considered as formalized measures of primary process mentation. An intensification of primary process mentation under psychedelics has been posited in contemporary psychoanalytic and neuroscientific literature (e.g., Carhart-Harris and Friston, 2010, 2019; Fischman, 2019; Lichtenstein and Hoeh, 2024; Guss, 2022; Buchborn et al., 2023; Koslowski et al., 2023).

To return briefly to the central theme of the present study, in Matte Blanco (1975) system of bi-logic, the primary and secondary processes are reformulated through the substrate of logic: “the principle of symmetry subtends, and thereby is able to account for, all the characteristics of the primary process” (Grotstein, 2000, p. 61). To be clear, the symmetrical mode of being runs parallel to the primary process, and the asymmetrical mode of being to the secondary process. Given that primary process mentation has long been implicated in psychedelic altered states, and the symmetrical mode is a reformulation of the primary process, it is no great leap to postulate that psychedelics initiate a shift from the asymmetrical to the symmetrical mode.

## 4 Contemporary psychoanalytic thought on psychedelic action

In this section, I summarize the psychodynamic and neuropsychoanalytic processes which have been commonly described by contemporary researchers studying psychedelic altered states. Categorization is for descriptive purposes only - it is recognized that these processes are continuous, free-flowing, and generally inseparable in experience.

### 4.1 Ego dissolution and defense reduction

A significant psychodynamic process observed in early psycholytic studies was the reduction and dissolution of ego defenses (e.g., Eisner and Cohen, 1958; Abramson, 1967; Sandison et al., 1954), and ego dissolution has often been viewed as an essential mechanism of transformation in psychedelic therapy (Buchborn et al., 2023). Psychedelics are thought to facilitate the dissolution and re-constitution of the subject’s object relations, relaxing entrenched defense mechanisms which have formed around one’s prior relational experience (Fischman, 2019; Buchborn et al., 2023). Fischman (2019) views the ego itself as an overarching system of defenses which regulates object relations and their significance for the self. Psychedelics are thought to de-activate the defenses which “help the ego moderate the threat of losing the loved object” (Fischman, 2019, p. 53).

A common experience in psychedelic therapy is the diminishing of self-identifications. At lower doses, e.g., in psycholytic therapy, the effect is more subtle (Passie et al., 2022). The autobiographical self “gradually softens and cedes its prominence as the organizer and creator of present experience” (Guss, 2022, p. 456). This is equivalent to an increased state of being and becoming, as opposed to conscious thinking and reflection, felt as a greater sense of connectedness and acceptance. The noetic quality of psychedelic altered states is attributable to the notion that the dissolution of one’s defensive structures feels somehow more real - “realer than real” - a childlike state of wonder in which the subject feels that they are experiencing the world anew (Fischman, 2022, p. 2). At higher doses, self-dissolution can occur in its entirety, being felt as a sense of mystical unity and boundless love or terror, thrusting one into the oceanic, omnipotent, or helpless feelings of infancy (Fischman, 2019, 2022; Lichtenstein and Hoeh, 2024).

Psychedelics are also thought to initiate the re-organization of *self-states*, the idea that the ego is formed of a multiplicity of distinct selves with competing drives and relational constitutions (Bromberg, 2009). Considering Bromberg’s (2009) notion that self-states may be dissociated, in conflict, or poorly integrated, Guss (2022) holds that psychedelics “reliably perturb the mechanism by which conventional identity and self-recognition are maintained,” resulting in a state in which “disparate self-states communicate and share co-consciousness with one another” (Guss, 2022, p. 457). This allows the psychedelic user to arrive at a self-awareness which seems oddly familiar but long forgotten. The return of previously dissociated self-states can often result in feelings of joy, love, and gratitude, as well as helplessness, discomfort, and dread, as repressed memories and emotions return to the surface, equivalent to abreaction in the Freudian lexicon (Breuer and Freud, 1893). This psychical process can be thought to be reflected somatically in a ‘purge’, in which contents are violently expelled from the gut, this phenomenon regarded in shamanic traditions as of integral therapeutic value (Fotiou and Gearin, 2019).

### 4.2 Regression

The contemporary psychoanalytic literature has also emphasized the importance of regression in the context of the therapeutic use of psychedelics (Barrett, 2022; Lichtenstein and Hoeh, 2024; Fischman, 2019, 2022; Rundel, 2022; Buchborn et al., 2023). Fischman (2019) holds that primary process mentation manifests in psychedelic altered states by way of a regression to early relational experiences. One regresses to a state of openness preceding when one’s defenses were formed: “perception unprejudiced by habitual expectation” (Fischman, 2019, p. 54). Such experiences can vary between the highly negatively valenced, where one is thrust into states of relational deprivation, painful or traumatic memories, childlike feelings of messiness, and overwhelming dependence; and expansive states of connectivity, meaning, and significance. According to Rundel

psychedelic work can facilitate regression to points of trauma and developmental rupture, as well as to unrepresented preverbal and prenatal experience and thereby offer a greater capability for repair and recovery than is available in our more conventional methods for analytic healing. (Rundel, 2022, p. 478)

One might think of this regression along Winnicott lines. If psychical development was interrupted at an early stage of one’s life due to trauma or environmental failure, it might be necessary to regress to that point of interruption in order that one may arrive at “a new progression of the individual process which had stopped,” culminating in a “return from regression to dependence, in orderly progress towards independence” (Winnicott, 1955, p. 22). Barrett views psychedelic therapy as offering the potential to “safely experience a felt sense of early failures in the facilitating environment” (Barrett, 2022, p. 488). The flip side of this is the potential for re-traumatization if one does not have a safe psychical or physical transitional space in which to move through disturbing early relational experiences.

### 4.3 Imagery, symbolization, archetypes, and free association

The de-activation of ego defenses is associated with unconstrained primary process mentation, manifesting in an intensification of dream-like imagery, symbolization, metaphor, and free association, aligning with an increased salience in sensory information, giving “a new meaning to previously disregarded aspects of external objects and internal representations” (Fischman, 2019, p. 55). Guss (2022) describes the potential for psychedelics to promote ‘hyper-associative’ states of free association by reducing both voluntary and automatic constrains on thought and speech. This is felt as “a reduction in the generation of familiar narratives along with a rush of unusual, unexpected content, all of which can lead to freer associations and enhanced imagination and creativity in a type of thinking Freud dubbed primary process” (Guss, 2022, p. 462). Guss links this process to an increase in transference relations. This is context dependent: identifications are projected onto the therapist in psychedelic therapy, onto the shaman in shamanistic practice, or onto the medicine itself. For example, ayahuasca is commonly experienced as *grandmother*, an archetypal Great Mother (Funder, 2021).

Experiences of archetypal symbols and dream-like narratives conforming with Jungian archetypes (Jung, 1959/1991) often manifest in psychedelic altered states (Grof, 2009; Clark, 2021; Richards, 2015). Therianthropic (animalistic) transformations, symbols of masculine and feminine, archaic script, and encounters with archetypal figures are all commonly reported, tending to “evoke archetypal and spiritual experiences related to our species-specific evolutionary development” (Carhart-Harris, 2007, p. 184). Grof states that “the subject can identify with the role of the Mother, Father, Child, Woman, Man or Lover. Many universalized roles are felt as sacred” (2009, p. 202).

### 4.4 Psychedelic neuropsychoanalysis: the REBUS model

A classic view of psychedelics is that they open the ‘doors of perception’^4^ by facilitating an expansion of experience (Huxley, 1954/2004). According to this notion, the brain generally serves an inhibitory function, *suppressing* experience rather than generating it:

Each one of us is potentially Mind at Large. But in so far as we are animals, our business is at all costs to survive. To make biological survival possible, Mind at Large has to be funneled through the reducing valve of the brain and nervous system. (Huxley, 1954/2004, p. 11)

As Huxley intuits, biological organisms must conserve energy to survive. Friston’s (2010) *free energy principle* (FEP) holds that organisms do this by making top-down predictions about bottom-up sources of information: external (sensory, environmental) and internal (affective, homeostatic). These predictions are organized into a hierarchy, enabling one to increasingly rely on predictions rather than processing incoming information, thereby minimizing energy expenditure, also expressed in terms of entropy, uncertainty, and surprise (Friston, 2010). This predictive hierarchy is iteratively updated as errors are detected, equivalent to *reality testing* in the Freudian lexicon (Hohwy, 2013; Carhart-Harris and Friston, 2010). The relative salience afforded to predictions and bottom-up information is modulated by the degree of confidence placed in each within a given context. This framework aligns with the operation of the primary and secondary processes in Freud’s (1900, 1911, 1915) drive theory (Carhart-Harris and Friston, 2010; Bazan, 2023). The (top-down) secondary process generally suppresses the unbound energy of the (bottom-up) primary process through reality testing, this process being disrupted in altered states of consciousness (Carhart-Harris and Friston, 2010; Koslowski et al., 2023).

Applying the FEP framework, the *relaxed beliefs under psychedelics* (REBUS) model holds that psychedelics relax the salience afforded to top-down predictions, thereby increasing receptivity to novel (new and long-forgotten) sensory and affective information. This process is associated at a neurobiological level with disruption to *default mode network* functioning and the liberation of subcortical affective activity (Carhart-Harris and Friston, 2019). This framing is thought to be conceptually consistent with the psychedelic amplification of the psychodynamic processes outlined above in this section: ego dissolution, the de-activation of entrenched defenses, regression to early relational states, and intensified states of free association (e.g., Fischman, 2019; Koslowski et al., 2023; Buchborn et al., 2023; Guss, 2022). As described above, these phenomena are associated with the flattening of secondary process inhibition and the manifestation of primary process mentation.

## 5 A bi-logic account of psychedelic action

Freud was originally focused on the role of the instinctual drives and energy in psychical life. Matte Blanco shifts away from this emphasis by proposing a new frame of reference based on logico-mathematical principles, applying the system of formal logic elaborated by Russell and Whitehead (1910) in *Principia Mathematica* (1910) to his psychoanalytic understanding of mental functioning. This is a “phenomenological-psychoanalytical-logical” approach (Matte Blanco, 1975, p. 216).

At a cognitive level, the asymmetrical mode of being, Aristotelian logic dominates (Matte Blanco, 1975). One differentiates between individual objects by implicit categorization into sets and classes. Experience is shaped by relations in space and time, and opposites contradict each other. This is the form of logic we are familiar with and use to theorize - asymmetrical logic is essential even to conceiving of other modes of psychical functioning. At a deeper affective level, the psyche is increasingly governed by symmetrical relations and the symmetrical mode of being. The symmetrical mode of being is characterized by symmetry, unity, generalization, spacelessness, timelessness, an absence of negation and contradiction, and boundless affective experience. These principles provide a logical basis for the characteristics of unconscious processes identified by Freud (1900, 1911, 1915): absence of time and negation, displacement, the primacy of psychical over external reality, and a lack of mutual contradiction and condensation.

Matte Blanco observed symmetrical thinking to be latently prevalent in his work with psychotic patients, his clinical experience mutually informing the development of his metapsychology. However, he held that a degree of symmetrical thinking is exhibited in all people, particularly in dreams, fantasies, and states of intense emotion. The distinction between these logical systems is fluid; they are always at interplay. Whereas Freud saw the operation of the primary and secondary processes as operating in dialectical conflict, the symmetrical and asymmetrical modes of being are “inescapably conjoined and operate reciprocally in an irreducible binary opposition” (Grotstein, 2000, p. 69). This is to say that other than at the extremes, any given psychical state involves a degree of both asymmetrical and symmetrical relations.

In the following sub-sections, I outline the principles of symmetrical logic and relate them to psychedelic altered states, referring to extracts describing psychedelic phenomenology to illustrate these principles. The purpose of these extracts is illustrative and anecdotal: they are intended to describe concepts and not to present empirical evidence for the account presented here, which will demand further empirical study. I will keep the expression of these concepts in terms of logico-mathematical symbols to a minimum, preferring to describe them in their most digestible form insofar as this maintains the substance of the description.

For the sake of clarity, this framework describes in philosophical terms one aspect of mental functioning (logic) which is thought to be useful in conceptualizing a range of experiences. It tends towards the philosophical, heuristic, phenomenological, and literary over the scientific, and is not in any way intended to be comprehensive in describing mental functioning.

### 5.1 The principle of symmetry

The *principle of symmetry* holds that asymmetrical relations are treated as if they were symmetrical. This means that *the converse of a relation is treated as identical to that relation*. For example, John is Peter’s father. This is an asymmetrical relationship, given that by Aristotelian logic, the converse would amount to an absurdity. But according to the principle of symmetry, “if John is the father of Peter, then Peter is the father of John” (Matte Blanco, 1975, p. 38). As stated above, Matte Blanco commonly observed this form of thinking with his schizophrenic patients. Here is a personal anecdote of this kind of thinking in the psychiatric context:

*A new doctor began work in the secure ward of a psychiatric hospital. A patient approached her and said: “you must be my patient; I am your new doctor.”*

Following the principle of symmetry (inadvertently), the patient concluded that if this was their new doctor, then they must also be a new doctor, and the doctor their patient.

Furthermore, pursuant to the principle of symmetry, the relation between the part and the whole is treated as if it were symmetrical. Thus, the part is *identical* to the whole and vice versa: “the total (and therefore the potentialities of the total) is included in any part, which results in any part being identical to the total, and in consequence identical also to any other part” (Matte Blanco, 1975, p. 39). We can imagine that Plotinus reports from a symmetrical state of consciousness when he says that

everything in the intelligible heavens is everywhere. Any thing is all things. The sun is all stars, and each star is all stars and the sun. (Plotinus, as cited in Borges, 1944/2000, p. 32)

An illustration of this principle in psychedelic altered states is the experience of oneself as identified or unified with an animate or inanimate object. Describing a mescaline experience at his home, Huxley reports that

I spent several minutes - or was it several centuries? - not merely gazing at those bamboo legs, but actually being them - or rather being myself in them; or, to be still more accurate (for “I” was not involved in the case, nor in a certain sense were “they”) being my Not-self in the Not-self which was the chair. (Huxley, 1954/2004, p. 10)

This is, of course, nonsensical by ordinary logical standards. But by symmetrical logic, Huxley and the bamboo legs are both part of the room, or part of the class of objects, inanimate and animate. Each part is identical to the whole and to each other part. Thus, Huxley *is* the chair legs. For Huxley, this apparently bizarre symmetrical apperception is of overwhelming and transcendent import. It is experienced as “the sacramental vision of reality… where everything shone with Inner Light, and was infinite in its significance” (p. 6). We can intuit that Huxley is awe-struck by the symmetrical mechanisms of mind (his own and ‘Mind at Large’) as they manifest in the dissolution of boundaries between self and other.

### 5.2 The principle of generalization

The *principle of generalization* treats

an individual thing (person, object, concept) as if it were a member or element of a set or class which contains other members; it treats this class as a subclass of a more general class, and this more general class as a subclass or subset of a still more general class, and so on. (Matte Blanco, 1975, p. 38)

At deeper levels of the symmetrical mode of being, one increasingly generalizes the characteristics of an individual to a class to which that individual belongs. In dream states, the principle of generalization is consonant with *condensation*, the notion that one figure can represent multiple others in the dreamer’s life (Freud, 1900; Matte Blanco, 1975). Another manifestation of this principle is *transference*. For example, a patient treats their analyst, Ms. A, as if she were the patient’s mother, Ms. M, by virtue of the fact that they are members of the same class: the class of women. Similarly, one might experience their mother as belonging to “the class of women who feed materially” and a teacher as belonging to “the class of men who feed mentally” (Matte Blanco, 1975, p. 42). At a deeper unconscious level, one might generalize further, experiencing the teacher as a mother who feeds, thereby “treating both classes as subclasses of a more general class, that of those who feed, either materially or mentally” (Matte Blanco, 1975, p. 42). These examples describe a distorted perception of the real human who is the object of the generalization (or transference), who maintains their central identity but loses characteristics of their individuality.

Describing a psychedelic experience in which he sits outside, eating homemade bread made by one of his friends, Alan Watts reports that “it tastes like the Original Bread of which mother’s own bread was a bungled imitation.” He experiences his friend as “a creature of all ages, baby, moppet, maid, matron, crone and corpse, evoking love of all ages” (Watts, 1962/2013, p. 45). Each of Watts’s friends appear “as immortal archetypes of themselves without, however, losing their humanity” (Watts, 1962/2013, p. 44).

Here, the propensity for symmetrical logic to manifest in generalized archetypal and transpersonal experiences is evident. Matte Blanco does not deal explicitly with the relation of his work to Jung’s concepts of the archetype and the collective unconscious, but the parallels are manifest. Watts experiences his friends as individuals, but also as members of a more general class: the class of lovely creatures, young and old. The operation of the principles of symmetry and generalization endow Watt’s friends with a sacred dimensionality because they are experienced with transcendent and timeless qualities. Similarly, Grof (2009) holds that in situations of ‘group consciousness’ induced by psychedelics, the subject can feel “simultaneously identified with all individual members of a particular group; these archetypical experiences represent personified concepts of the roles involved” (2009, p. 202). For instance, one might identify with the struggle of their ancestors or with the group in which they are engaging in ceremony (Richards, 2015). In these situations, the psychedelic user generalizes their own experience with that of a class, or vice versa.

### 5.3 Timelessness and spacelessness

The operation of the principle of symmetry gives rise to *the progressive dissolution of relations in time and space*. If event *y* is followed in time by event *x*, pursuant to symmetrical logic this entails the converse: event *x* is followed by event *y*. Succession in time is inconceivable when symmetrical logic reigns, and the same is true of spatial relations (Matte Blanco, 1975). Spatial–temporal relations require a distinction between the part and the whole, a serial ordination. By symmetrical logic, ‘a minute is part of an hour’ entails that ‘an hour is part of a minute’, and ‘a page is part of a book’ entails that ‘the book is part of the page’. Thus, relations in time and space are rendered void and meaningless.

Space and time are commonly felt to be transcended in psychedelic altered states (Richards, 2015; Letheby, 2021; Griffiths et al., 2006). Richards holds that

people who have mystical experiences often claim not only that they were distracted or unaware of the passing of time, but that the state of consciousness they were experiencing was intuitively felt to be “outside of time”… space simply seems to be a concept that works well as we think and function in the everyday world of sense perception, but that somehow is either incorporated or left behind in the eternal realms. (Richards, 2015, p. 71)

A British MP, Christopher Mayhew, took mescaline live on television in 1955. He reported that

I remembered the afternoon not as so many hours spent in my drawing room interrupted by these kind of excursions [a state of complete bliss], but as countless years of complete bliss interrupted by short spells in my drawing room. (as cited in Letheby, 2021, p. 46)

Watts, describing a psychedelic experience in California, reports that

time is so slow as to be a kind of eternity, and the flavor of eternity transfers itself to the hills - burnished mountains which I seem to remember from an immeasurably distant past, at once so unfamiliar as to be exotic and yet as familiar as my own hand. (Watts, 1962/2013, p. 30)

Spatial experiences often evoke Alice in Wonderland, an expansion or contraction of one’s body in relation to one’s environment (Letheby, 2021). One patient

reported that he felt himself to be six inches in height. Curiously the objects in the room underwent a similar and proportionate transformation, while the guide and another observer retained their normal dimensions, appearing to him to be giants. (Masters and Houston, 1966/2000, p. 70)

A subject in the ‘Good Friday Experiment’ stated that “Matter and time seemed to be of no consequence. I was living in the most beautiful reality I had ever known, and it was eternal” (Pahnke, 1963, p. 144).

### 5.4 Absence of mutual negation and contradiction: the unity of opposites

*Absence of mutual negation and contradiction* is a characteristic of the unconscious which dictates that two competing drives can be simultaneously present at an unconscious level without cancelling each other out (Freud, 1915). By the principle of symmetry, *p* and *not-p* are treated as *identical*, so they do not negate or contradict (Matte Blanco, 1975). A well-known example is love and hate, which coexist at an unconscious level. One can alternate between these states of mind, but it takes a degree of psychical development to *simultaneously experience* love and hate for a particular object (e.g., Klein, 1946).

Sandison et al. (1954) report a case of psycholytic therapy using LSD. This patient

saw two women as hallucinations, one middle-aged, large, coarse, dressed in an unpleasant and untidy purple dress, and having a revolting smell. The other was a beautiful woman dressed in the style of the French court before the revolution. She alternately became both of these women, seeing herself at first as a loathsome harlot and then as a wished-for lady of beauty, elegance, and position. This experience agreed well with her biographical details and helped her greatly to understand her position. (Sandison et al., 1954, p. 495)

A possible interpretation suggested by this anecdote is that, at deeper levels of this patient’s mind, she is both good and bad, clean and dirty, desired and disgusting. Only with the conscious realization that she is, in essence, a unity of opposites, a multiplicity of self-states (Bromberg, 2009), can she come to a greater sense of self-understanding and acceptance, at least if only temporarily.

The unity of opposites and the dissolution of self-other boundaries are experiences commonly reported in psychedelic altered states (e.g., Pahnke, 1963; Griffiths et al., 2006). Pahnke observes that

In the experience of internal unity there is a loss of empirical content in an empty unity which is at the same time full and complete… The “I” both exists and does not exist. External unity is experienced through the empirical multiplicity of the external world with the insight that all is One. (Pahnke, 1963, p. 70)

In Buddhist teachings, the concept of *emptiness* signifies the insight that self and phenomena are devoid of innate existence (Van Gordon et al., 2017). This is also translated as *boundlessness*, suggesting that one cannot intrinsically draw boundaries between phenomena, including self and other. Both in contemplative and psychedelic altered states, the absence of mutual negation and contradiction allows for *paradox*, whereby the internal world can be simultaneously empty and full, the external world both multiplicitous and unitary.

### 5.5 Infinity, terror, and the expansion of emotional depth

In the bi-logic framework, the symmetrical mode of being corresponds with affect. A general rule is “the greater the degree of symmetry, the greater the magnitude of the affects” (Matte Blanco, 1975, p. 174). Matte Blanco holds that

At each [unconscious] level there is a given magnitude of each of the variables of an emotion (intensity, tension, frequency, capacity). At the superficial levels, each of these values or magnitudes is small; it increases as symmetrical relations increase, and then becomes infinite. As the level gets still deeper, space–time begins to disappear, and also discrete elements; the infinites fuse into a tranquility, where there is no emotion but only being. All this appears a necessary and clear consequence of the present conception. But it is shrouded in mystery. (Matte Blanco, 1975, p. 175)

Emotional states under psychedelics are often highly negatively valenced or challenging, especially in the ‘come up’ phase of the experience (Brouwer et al., 2025). Early studies describe intense experiences of dissociation, fear, and depersonalization (Cohen, 1960). This has been replicated in recent studies demonstrating a significant increase in anxiety, fear, loneliness, and paranoia during psychedelic altered states (Carbonaro et al., 2016; Carbonaro et al., 2016; Griffiths et al., 2006; Barrett et al., 2016). One might think of such experiences in terms of a “fear of symmetry” (Grotstein, 2000, p. 64), alluding to Blake’s classic poem “The Tyger,” or as “the fear of unneutralized, ever-proliferating infinity” (Grotstein, 2000, p. 63).

Above, I have outlined the hypothesis that psychedelics act to disrupt top-down inhibition of *entropic* subcortical (affective) activity (section “*Psychedelic neurospsychoanalysis: the REBUS model*”). The relaxation of beliefs under psychedelics is susceptible to provoke a *terror of entropy*, infinite entropy, a state in which one senses an internal mode of being which is entirely incompatible with biological life. In the language of Winnicott, this is a “primitive agony,” evoking the terror of “falling forever,” or the “return to an unintegrated state” (Winnicott, 1974, p. 103). If the psychedelic state were to persist indefinitely, this fear would be actualized, given that one would be psychotic, and would in fact be incapable of living their life. In this state of intense anxiety, one becomes fearful of overwhelm by unregulated affect, felt as a total loss of control and helplessness which will, it is imagined, last forever.

Although such a state involves risks of trauma or re-traumatization (Evans et al., 2023), ‘bad trips’, when they do occur, do not frequently persist beyond the acute phase of the drug’s effects (Yao et al., 2024; Gashi et al., 2021). Moreover, ‘challenging psychedelic experience’ might be a better framing, given that negatively valanced psychedelic experiences are positively correlated with enduring positive benefits to well-being (Carbonaro et al., 2016). Following the early challenging stages of psychedelic experiences, emotional breakthroughs often occur (Roseman et al., 2019). For instance, a Jewish psilocybin user who had, for as long as he could remember, felt a sense of disconnection from his community, reports that

I was able to feel the agony of centuries of persecution, alienation and isolation, of being the stranger in a strange land, and of the Holocaust… [Later in the experience] I understood, felt empathy for and accepted this pain in my heart. Instead of being overwhelmed, I felt expansion, ease and comfort in accepting my place in the scheme of things. (Metzner, 2004, p. 252)

Love can be an overwhelming experience, even in a sober state: “the loved one… appears as the summum of perfection and of what is desirable…love is felt as surpassing time…It also transcends space and distance” (Matte Blanco, 1975, p. 184). One feels “infinite degrees” of the characteristics of the beloved (Matte Blanco, 1975, p. 185). Such experiences are exacerbated in psychedelic altered states. A notable example of this type of symmetrical thinking is the common psychedelic experience of loving communion or identification with deities (Richards, 2015; Masters and Houston, 1966/2000). From a logical perspective, if ‘I am loving’ and ‘Jesus Christ is loving’, we are both members of the class of loving beings, and of beings in general. Hence, the claim that ‘I am Jesus Christ’. Such an insight is clearly risk-laden if one cannot move flexibly between the symmetrical and asymmetrical modes, and if one neglects to account for the notion that, by the same symmetrical logic, it is not only I who am Jesus Christ, but all beings. On this point, Matte Blanco holds that “when we assert ourselves as God and (try to) occupy the whole space we find that the same space may also be occupied by another God: in fact, an infinite number of them… we are smaller than anything conceivable” (Matte Blanco, 1981, as cited in Grotstein, 2000, p. 78).

### 5.6 The primacy of psychical over external reality

This is not strictly a logical principle in itself. Rather, it is a characteristic of symmetrical thinking which is necessitated by the principles of symmetry and generalization: “the unconscious treats both [psychical and external realities] as elements of the same class… then it treats the two as if they were identical” (1975, p. 43). Thus, by symmetrical logic, psychical reality is experienced as part of shared reality, i.e., *as if* it were objective.

Reports of psychedelic experiences often involve fantastical, dreamlike narratives which are projected onto the external world. The language of psychedelic reports tends to indicate the salience of one’s fantasies. For example, “my body began to dissolve into the soil, merging with the Earth… I sank down into the Earth and became a river, which flowed into an ocean” (Metzner, 2004, p. 230). In reporting dreams or fantasies, we do not tend to preface our description with concrete qualifiers such as “it felt like” or “I had a fantasy in which,” and this is particularly true of psychedelic experience, which tends to be particularly arresting. This is one of a plethora of anecdotes describing an experience which might be interpreted as representative of a psychical and relational process, arising in a transitional space between psychical and shared reality (Fischman, 2022). Here, the fantasy of dissolution into nature may symbolize this man’s connection with his own deeper nature or with his primary attachment figures.

Despite the salience of their fantasies, the psychedelic user generally keeps one foot in reality, knowing at some level that these fantasies are not publicly observable. Thus, the framing of psychedelics as *hallucinogens* is misleading, given that this term implies a lack of awareness that one is experiencing phenomena which are not publicly observable (Letheby, 2021; Carhart-Harris and Goodwin, 2017). It may be more accurate to say that one moves increasingly *closer* to the primacy of psychical over external reality at deeper levels of symmetrical thinking.

### 5.7 Symmetrical visual and sensory experience

The above discussion has focused on symmetry as it is phenomenologically represented through narrative structures and affective experience. Matte Blanco’s work only hints at the realms of symmetrical visual and sensory experience. Some well-known examples of such symmetrical manifestations in psychedelic phenomenology are multi-dimensional visual geometric patterns, repetition of archetypal symbols such as eyes or mandalas, a sense of oneness with music, and synesthesia (Makin et al., 2023; Richards, 2015; Metzner, 2004). We can intuit that the increased prevalence of symmetrical narrative logic induced by psychedelics correlates with, or is precluded by, the emergence into consciousness of symmetrical affective, visual, and sensory experience (McGovern et al., 2024).

### 5.8 The homogeneous indivisible totality

The homogeneous indivisible totality is a state absolutely dominated by symmetrical relations; it is the ultimate end and the logical conclusion of symmetrical thinking. In this state, no distinction is made between self and other such that “self and all other persons are one.” This state is “alien to the notion of death… something indivisible cannot die” and “alien to any notion of physical or symbolic space, physical or symbolic time” (Matte Blanco, 1975, p. 350). Such an experience has been described in psychedelic reports in terms of ineffability, oneness, mystical unity, religious experience, ego dissolution, and cosmic consciousness (e.g., Richards, 2015; Metzner, 2004; Ko et al., 2022; Griffiths et al., 2006).

A participant in a clinical trial of psilocybin reports that “There is no speck in the cosmos which is apart from this breath… I am seeing myself in everybody, and everybody in myself” (Richards, 2015, p. 62). An LSD subject suffering from alcoholism states that

it is very difficult to explain. There was nothing. It was like slowly drifting into empty space… I was God. God was me at that time - it seemed all men were here. We must truly all be one. I thought this is what has been said of eternal life. (Richards, 2015, p. 64)

Another LSD user “describes himself as having been caught up in an undifferentiated unity wherein the knower, the knowledge and the known are experienced as a single reality” (Masters and Houston, 1966/2000, p. 308). He reports that

I knew that the boundaries of my being now had been dissolved and that all other boundaries also were dissolving… What ‘I’ experienced in this ALL so far transcends my powers of description that to speak, as I must, of an ineffably rapturous Sweetness is an approximation not less feeble than if I were to describe a candle and so hope to capture with my words all of the blazing glory of the sun. (Masters and Houston, 1966/2000, p. 308)

These descriptions parallel attempts to parse the concept of the homogeneous indivisible totality through a mystical or religious cosmology (e.g., Grotstein, 2000; Bomford, 1999). It seems to be implicitly assumed in the psychoanalytic literature outlined above that mystical psychedelic experience is an emergent property of the other psychical processes identified above (e.g., Fischman, 2019; Lichtenstein and Hoeh, 2024; Buchborn et al., 2023). That is, mystical experience is arrived at when ego dissolution is complete, when one regresses beyond one’s earliest relational experience and into a state of boundless unity, love, terror, or oceanic feeling. The contribution of bi-logic theory is to conceive of this state not in the negative (e.g., the *absence* of ego functioning), or as a delusional wish fulfilment (Freud, 1927), but rather as a necessary and predictable consequence of the principles of symmetry and generalization.

It is thus maintained that bi-logic can serve to unite psychedelic altered states - from the psychodynamic to the transpersonal - on a continuum of logic which extends until the homogeneous indivisible totality at the depths of the symmetrical mode, facilitating a description on collective, transpersonal, or transcendental terms (Matte Blanco, 1975; Bomford, 1999; Grotstein, 2000; Rundel, 2022).

## 6 Discussion

I suggest that Matte Blanco arrives at a system for expressing psychical processes which can better serve to conceptualize psychedelic altered states along the lines of their apparently bizarre and illogical phenomenology. Although these states tend towards the mysterious and the ineffable, they also conform with an altered mode of being governed by an alternate logical structure which is ever-present and influential, though ordinarily far less accessible to conscious awareness: “the colossal base from which consciousness or asymmetrical being emerges” (Matte Blanco, 1975, p. 101). This apparent contradiction - between the ineffable and its expression in logical terms - breaks down when we consider the subjective qualities which the logical principles of the symmetrical mode of being represent: relationality, aliveness, messiness, paradox, emotion, unity, and mystery.

Importantly, it is anticipated that this framework can serve as a heuristic which can be utilized in the context of the therapeutic use of psychedelics. Matte Blanco (1975) views the general contribution of bi-logic theory to psychoanalytic clinical practice in terms of the modest aim of increasing understanding, thereby arriving “nearer to the inner self” of patients (p. 385). An enhanced understanding of psychedelic altered states informed by bi-logic theory will not result in a radically different therapeutic attitude, particularly assuming that we already aim to approach patient material with a stance of evenly suspended attention and negative capability.

However, in much the same way as psychedelics are thought to relax ingrained belief structures – opening to novel bottom-up information sources – new conceptual framings can offer clinicians a gateway to previously inconceivable communications from patients. Elucidating this point, Sjöstedt-Hughes (2023) argues for the utility of philosophical insight in integrating psychedelic altered states, conceptual framings facilitating an openness to the significance of these states and their enduring influence upon and commonality throughout traditions of philosophy and mysticism.

Presently, it is thought that bi-logic can serve as a heuristic container for reverie and “becoming at one” (Bion, 1962; Ogden, 2015, p. 294) with states of intense affect (from the terrifying to the wonderful), paradox, and the dissolution of self-other boundaries. It is precisely such disturbing and potentially transformative states which psychoanalytic clinicians are called upon to be receptive to and digest, ultimately contributing to the process of their integration. In this way, states which might otherwise be unthinkable can be stayed with and approached as universal aspects of human experience.

This is a paradox in itself. The theoretical framework is an abstraction of experience, a distancing, but it can also be utilized as a heuristic to feel into and come closer to the experience which the theory describes in philosophical terms. This same paradox applies to the relation between psychoanalytic theory and clinical practice more generally. The present contention, elaborated above, is that Matte Blanco’s framework is of marked utility in representing the experience of patients in psychedelic altered states, thereby serving in practice as a container in which to be with the human having this experience (oneself included) in a more intimate and empathic way.

I reiterate that there is nothing essential about the present conception, which is far from comprehensive in conceiving of psychedelic altered states and psychical functioning more generally. Other theoretical frameworks will be of greater utility to other clinicians in the face of the same underlying phenomena. Another alternative is to draw upon the work of Blake and the poets. All of these modes of enquiry - philosophical, psychoanalytic, neuroscientific, poetic - are thought to represent, with varying degrees of abstraction, a unitary underlying reality (Goldreich, 2025).

Importantly, a limitation of the bi-logic framework is that symmetrical logic is invariably expressed in terms of asymmetrical logic, given that the symmetrical mode of being is anathema to language and analysis (Matte Blanco, 1975). This means that, when describing the symmetrical mode of being, we are always operating from an additional degree of separation from the reality which is the subject of the representation. The same is true of poetry describing altered states, which may or may not be better at precipitating states of reverie and empathic relationality than psychoanalytic theory. Bi-logic is not poetic but, like poetry, it can serve as a container to get in touch with altered states through the filter - restrictive yet liberating - of language.

If psychoanalysis can attain an expanded comprehension of psychedelic phenomena by reference to bi-logic, this has a bearing on the question of whether psychoanalytic models of clinical practice can increasingly adapt to contain and work with these phenomena in the consulting room. This is a current issue, given that psychoanalytic clinicians have begun to re-integrate psychedelics into clinical practice, with new psychoanalytic case reports emerging on work with non-serotonergic (non-classic) psychedelics, such as ketamine (e.g., Rundel, 2022), which is sure to be followed by work with classic psychedelics as legal restrictions relax. Moreover, clinicians have made the case that the psychoanalytic container is particularly suitable for work with psychedelics, given the emphasis on psychodynamic processes in early psycholytic trials, and the frame of psychoanalytic psychotherapy, which is fastidiously designed to work with processes of regression, overwhelming emotion, and the altered states of free association, dreaming, and reverie (e.g., Guss, 2022; Rundel, 2022; Fischman, 2022). It is asserted that psychoanalytic clinical practice is also uniquely primed to work with symmetrical thinking, given that this mode underlies the psychodynamic processes described above.

Moreover, this discussion may be of consequence in terms of the dose which clinicians will feel able to work with. Psychoanalysis has historically been considered a suitable container for psycholytic therapy, but not high dose psychedelic therapy, in which transpersonal phenomena tend to emerge (Grof, 1973, 2009; Passie et al., 2022). This is consequential because there is a range of empirical evidence to support the view that the mystical, spiritual, and metaphysical aspects of psychedelic altered states modulate the therapeutic benefits observed in psychedelic clinical trials (e.g., Kangaslampi, 2023; Ko et al., 2022; Sjöstedt-Hughes, 2023), in line with Jung’s intuition that “the approach to the numinous is the real therapy, and inasmuch as you attain the numinous experience you are released from the curse of pathology” (Jung, 1973, p. 377; Walsh and Grob, 2006). Thus, if psychoanalytic theory can arrive at an expanded understanding of psychedelic altered states which overlaps with mystical and spiritual perspectives, as outlined above (e.g., section “*The homogeneous indivisible totality*”), this can serve to open new dialogues between practitioners of high and low dose psychedelic therapies.

A range of further research might be undertaken to subject the account I have outlined here to greater scrutiny. For instance, symmetrical logic might be operationalized based on an inventory which can be used to measure the prevalence of symmetry underlying different categories of experience: affective, sensory, and narrative. Such a measure could be used to test the hypothesis that psychedelics increase the prevalence of symmetry compared with ordinary states of consciousness. Moreover, a thematic analysis could be used to unpack the symmetrical qualities of the phenomenology of psychedelic states in a more comprehensive and rigorous way than there has been scope for in the present study.

## 7 Conclusion

In this conceptual study, I have reviewed classical and contemporary psychoanalytic models of psychedelic action and elaborated a novel ‘bi-logic account’, which posits that psychedelic altered states of consciousness increasingly tend towards the symmetrical mode of being. That is, psychedelics shift the balance between the asymmetrical and symmetrical modes such that the symmetrical mode is increasingly prevalent in subjective experience and influential in mental functioning compared with ordinary states of consciousness. Through this frame of reference, the psychedelic phenomena described above, such as experiences of intense affect, relationality, paradox, unity, the terror of entropy, and the dissolution of spatial–temporal relations, can be interpreted not in the negative - merely as the reduction of ego functioning, the relaxation of beliefs, or as illogical or miraculous aberrations - but rather as ever-present aspects of mental functioning which are made manifest in psychedelic altered states.

I have discussed a range of clinical implications of this framing in relation to the therapeutic use of psychedelics, particularly in the context of psychoanalytically-informed psychedelic therapy. Bi-logic is conceived of as a heuristic which can facilitate greater understanding of and empathy towards those in the midst of psychedelic experiences, contributing to a conceptual container which facilitates a greater receptivity to the states which the theory expresses in philosophical terms.

## Data Availability

The original contributions presented in the study are included in the article/supplementary material, further inquiries can be directed to the corresponding author/s.

## Footnotes

^1^Regressive perinatal experience also has a psychoanalytic basis if one considers Rank’s (1929) theory of birth trauma to be within the scope of psychoanalytic metapsychology.

^2^The ‘psychedelic renaissance’ refers to the revival of the mid-20^th^ Century cultural and research interest in psychedelics (Sessa, 2018). However, such a framing implies that psychedelic use began in the 1950s. Hallucinogenic plants have been used by humans for millennia - “since earliest man’s experimentation with his ambient vegetation” (Schultes et al., 1992, p. 9). Indigenous cultures use a vast variety of psychoactive regional plants as sacraments in religious rites, for divination, in rites of passage, to commune with spirits, and for holistic healing practices (Schultes et al., 1992).

^3^Although it is generally the case that primary and secondary process mentation map onto Freud’s distinction between unconscious and conscious, respectively, both processes can, to varying degrees, take place unconsciously or consciously (Bazan, 2023).

^4^Like ‘fearful symmetry’, the ‘doors of perception’ is a phrase which originates with William Blake. Blake writes that “If the doors of perception were cleansed, everything would appear to man as it is, infinite” (Blake, 1790/2000, p. 120). Moreover, Blake anticipates Freud and Friston when he states in his poem ‘The Marriage of Heaven and Hell’ that “*Energy is the only life and is from the Body and Reason is the bound or outward circumference of Energy* [emphasis added]” (p. 110).
